# Supplementary material for: Defining the molecular mechanisms of the mitochondrial permeability transition through genetic manipulation of F-ATP synthase
Source: Nat Commun. 2021 Aug 10;12:4835. doi: 10.1038/s41467-021-25161-x (PMC8355262; doi:10.1038/s41467-021-25161-x)
Supplement: Supplementary file 1 — Supplementary information. [file 41467_2021_25161_MOESM1_ESM.pdf]

SUPPLEMENTARY INFORMATION FOR

# Defining the molecular mechanisms of the mitochondrial permeability transition through genetic manipulation of F-ATP synthase

Andrea Carrer, Ludovica Tommasin, Justina Šileikytė, Francesco Ciscato, Riccardo Filadi, Andrea Urbani, Michael Forte, Andrea Rasola, Ildikò Szabò, Michela Carraro, Paolo Bernardi

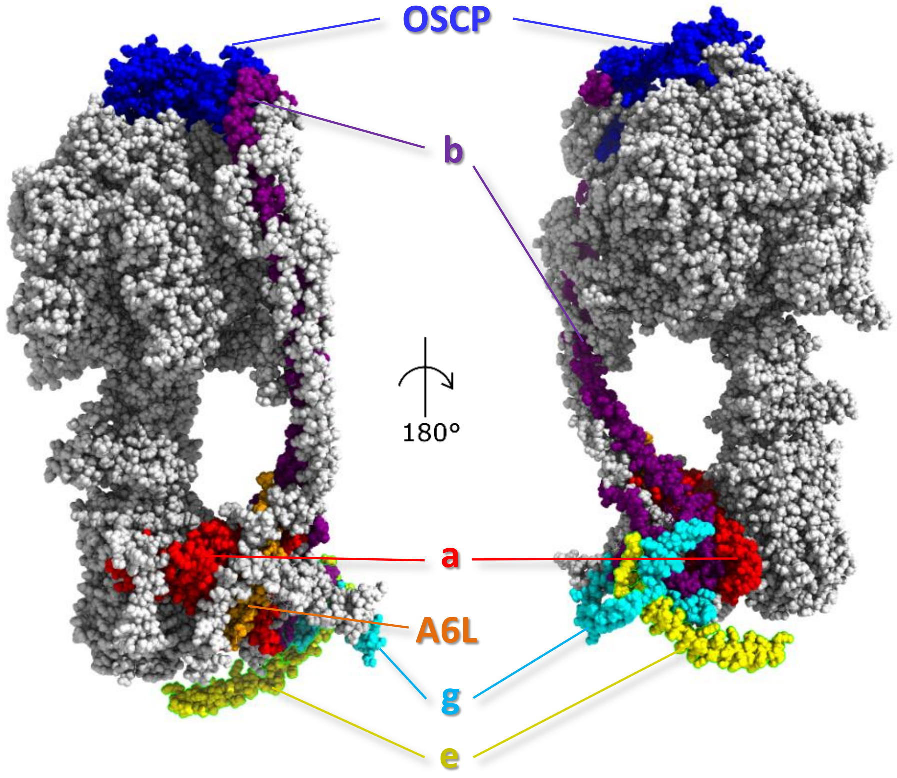


Supplementary Figure 1. **Structure of F-ATP synthase.** F-ATP synthase monomer (modified from Spikes et al.[1]), with highlighted subunits of interest.

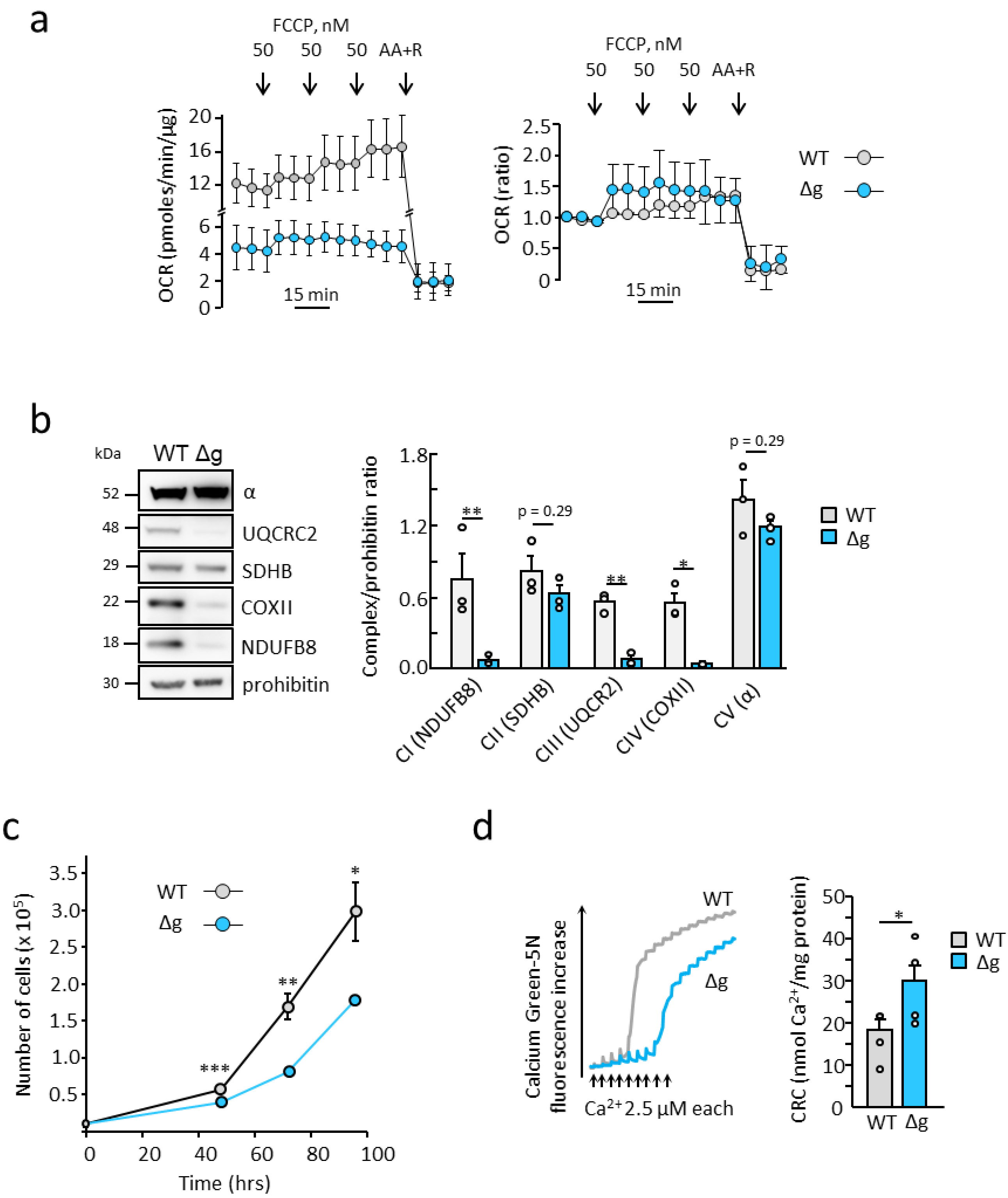
a
FCCP, nM
50
50
50
AA+R
OCR (pmoles/min/μg)
20
16
12
6
4
2
0
15 min
OCR (ratio)
2.5
2.0
1.5
1.0
0.5
0
WT
Δg
b
kDa
WT Δg
52
48
29
22
18
30
α
UQCRC2
SDHB
COXII
NDUFB8
prohibitin
Complex/prohibitin ratio
1.8
1.2
0.6
0.0
**
p = 0.29
**
*
p = 0.29
CI (NDUFB8)
CII (SDHB)
CIII (UQCR2)
CIV (COXII)
CV (α)
WT
Δg
c
Number of cells (x 10^5)
3.5
3.0
2.5
2.0
1.5
1.0
0.5
0
20
40
60
80
100
Time (hrs)
***
**
*
WT
Δg
d
Calcium Green-5N fluorescence increase
WT
Δg
Ca2+ 2.5 μM each
CRC (nmol Ca2+/mg protein)
50
40
30
20
10
0
*
WT
Δg

Supplementary Figure 2. **Oxygen consumption rate, oxidative phosphorylation complexes, growth rate and $Ca^{2+}$ retention capacity of HeLa-Δg cells**. **a** Effect of different FCCP concentrations on oxygen consumption rate (OCR) in wild-type (WT) and HeLa-Δg cells. Traces are average of three independent experiments. Left, absolute OCR values normalized to µg of protein; right, OCR was normalized to the initial value. **b** Western blot analysis of mitochondria isolated from WT and Δg cells for the indicated subunits of the OXPHOS system. Histogram refers to protein levels relative to prohibitin and represents the mean ± SEM of three independent blots, *$p<0.05$, **$p<0.01$ Two-sided Student's t-test. **c** Growth curve of WT and HeLa-Δg cells. Cells were counted 48, 72 and 96 hours after seeding. Data are expressed as the mean ± SEM of three independent experiments, *$p<0.05$, **$p<0.01$, ***$p<0.001$, Two-sided Student's t-test. **d** $Ca^{2+}$ retention capacity (CRC) of mitochondria isolated from WT (gray trace) and Δg cells (cyan trace). Representative traces are shown. Histogram refers to the nmol of $Ca^{2+}$ per mg of protein accumulated before $Ca^{2+}$ release and are mean ± SEM of five independent experiments, *$p<0.05$, Two-sided Student's t-test. Source data are provided as Source Data file.

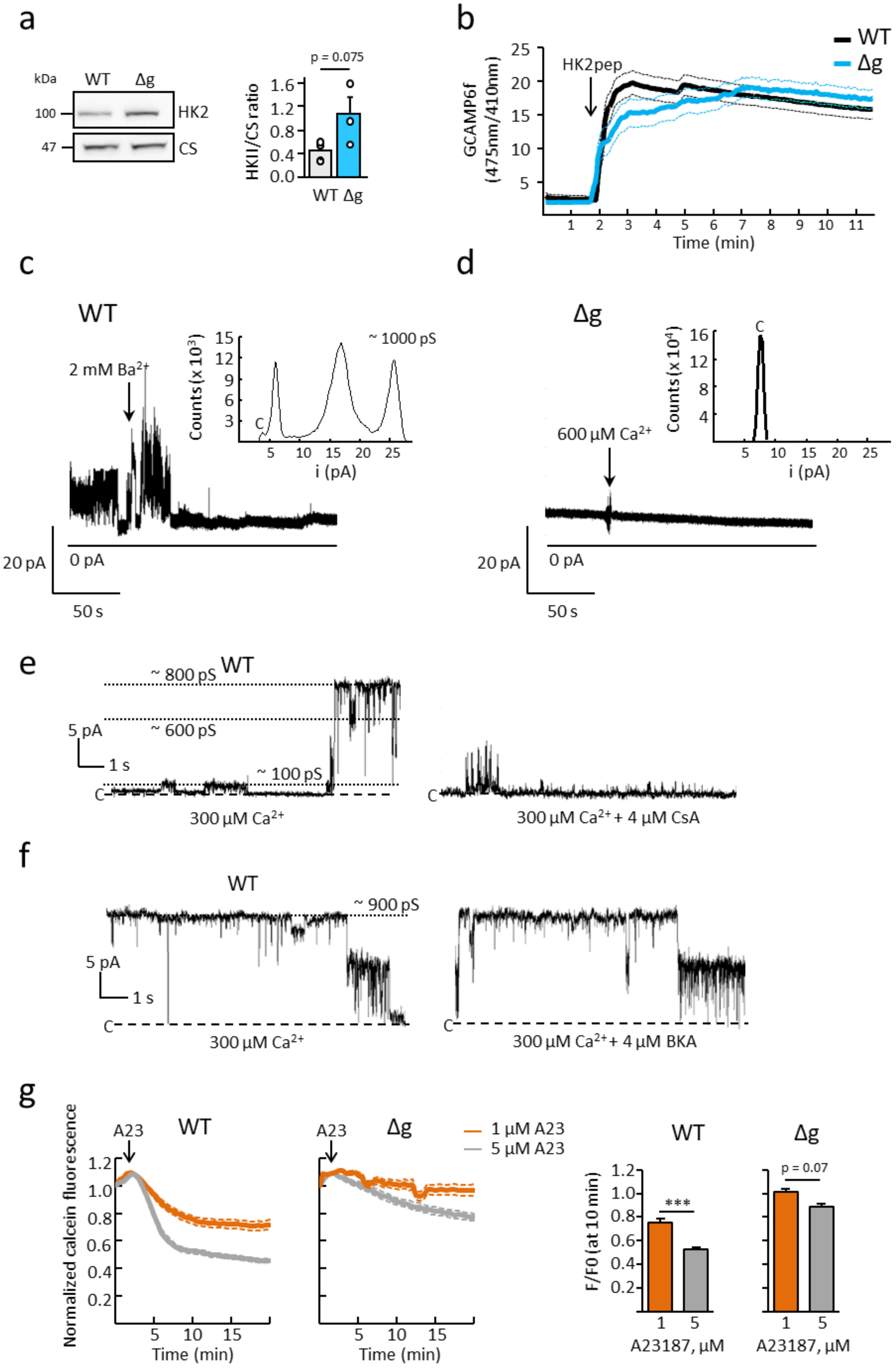
a
kDa
WT
Δg
100
47
HK2
CS
p = 0.075
HKII/CS ratio
1.6
1.2
0.8
0.4
0.0
WT Δg
b
WT
Δg
HK2pep
GCAMP6f
(475nm/410nm)
25
20
15
10
5
1 2 3 4 5 6 7 8 9 10 11
Time (min)
c
WT
2 mM Ba2+
Counts (x 10^3)
15
12
9
6
3
C
~ 1000 pS
5 10 15 20 25
i (pA)
20 pA
0 pA
50 s
d
Δg
600 μM Ca2+
Counts (x 10^4)
16
12
8
4
C
5 10 15 20 25
i (pA)
20 pA
0 pA
50 s
e
~ 800 pS
WT
~ 600 pS
5 pA
1 s
~ 100 pS
C
300 μM Ca2+
300 μM Ca2+ + 4 μM CsA
f
WT
~ 900 pS
5 pA
1 s
C
300 μM Ca2+
300 μM Ca2+ + 4 μM BKA
g
Normalized calcein fluorescence
A23
WT
1.2
1.0
0.8
0.6
0.4
0.2
5 10 15
Time (min)
A23
Δg
1 μM A23
5 μM A23
5 10 15
Time (min)
WT
Δg
F/F0 (at 10 min)
1.2
1.0
0.8
0.6
0.4
0.2
***
p = 0.07
1 5
A23187, μM
1 5
A23187, μM

Supplementary Figure 3. **Features of the permeability transition in HeLa-Δg cells and mitoplasts**. **a** Western blot analysis of hexokinase 2 (HK2) expression in isolated mitochondria. The histogram refers to the levels of HK2 relative to citrate synthase (CS) and data are mean ± SEM of at four (WT) or three (Δg) independent blots, p=0.075, Two-sided Student's t-test. **b** Changes in mitochondrial matrix $Ca^{2+}$ concentration of WT (black symbols) and HeLa-Δg cells (cyan symbols) measured by fluorescence changes of mitochondrially-targeted GCaMP6f. Where indicated 2.5 µM HK2 peptide (pep) was added. Data are average of 21 for WT and 30 for Δg single cell signals, and SEM for each time point is denoted by thin lines. Differences between WT and HeLa-Δg cells were not statistically significant at any time point, as assessed by Two-sided Student's t-test. **c** Extended (200 sec) wild-type current traces from which the recording of Fig. 2c, left panel was obtained. The bath contained 300 µM $Ca^{2+}$; where indicated, 2 mM $Ba^{2+}$ was added. Amplitude histogram was calculated in a time interval of 60 seconds before the addition of 2 mM $Ba^{2+}$, c, closed. **d** Extended (200 sec) Δg current traces from which the recording of Fig. 2c, right panel was obtained. The bath contained 300 µM $Ca^{2+}$; where indicated, 600 µM $Ca^{2+}$ was added. Amplitude histogram was calculated in a time interval of 60 seconds before the addition of $Ca^{2+}$; c, closed. **e,f** Representative current traces showing PTP channel activity obtained by patch-clamping isolated mitoplast from HeLa WT cells in the presence of 300 µM $Ca^{2+}$($V_h$ = +20 mV) and of CsA and BKA, respectively. **g** Calcein release in HeLa cells upon addition of 1 µM (orange line) or 5 µM (gray line) A23187. Data are mean ± SEM of 88 ROIs over four independent experiments (for 1 µM A23187) or 41 ROIs over three independent experiments (for 5 µM A23187) for HeLa WT cells and of 27 ROIs over two independent experiments (for 1 µM A23187) or 158 ROIs over eight independent experiments (for 5 µM A23187) for HeLa Δg cells. Histograms refer to calcein fluorescence after 8 min of the addition of the indicated concentrations of A23187, and represent the average ± SEM of the above-mentioned ROIs, ***p<0.001, Two-sided Student's t-test. Source data are provided as Source Data file.

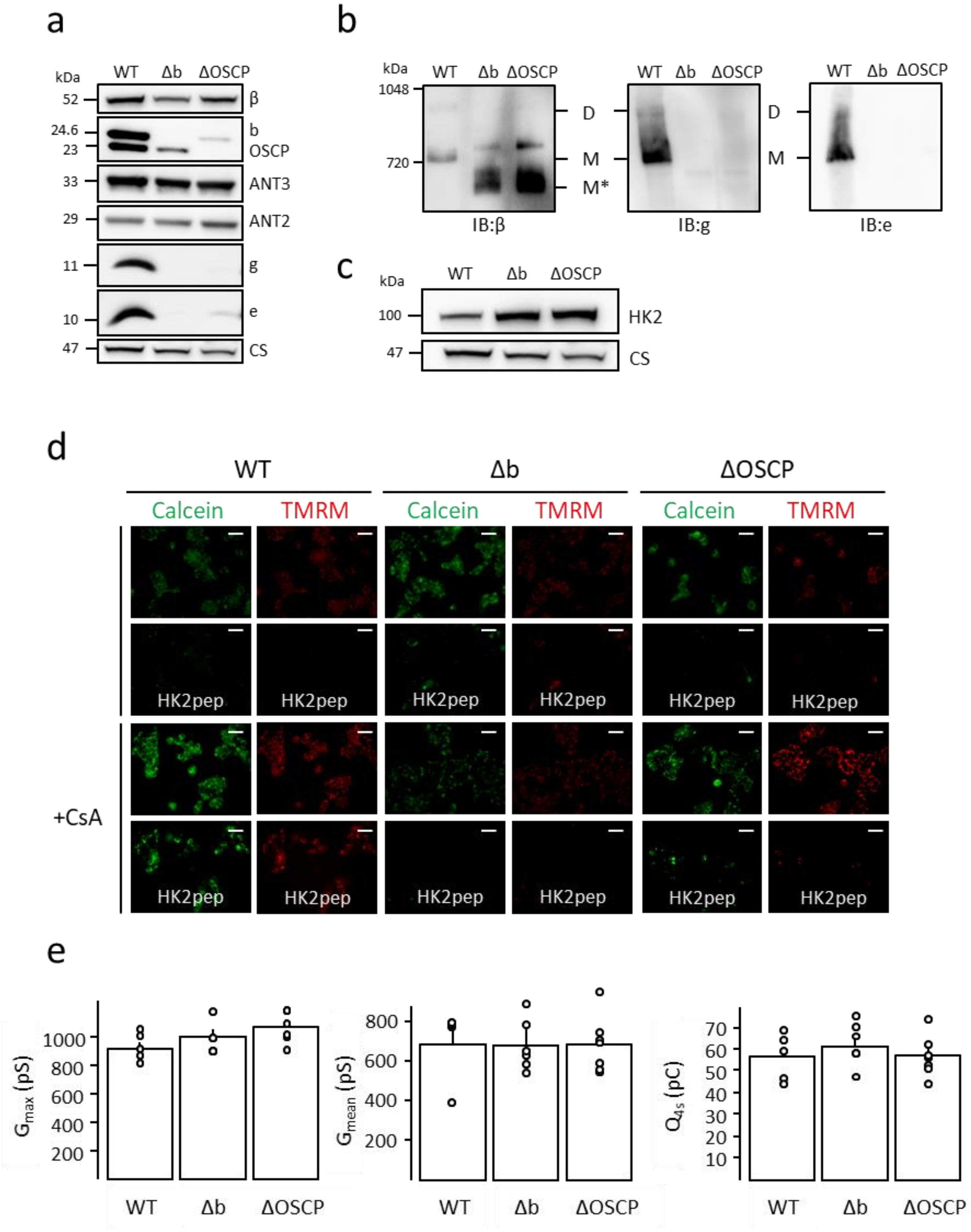
a
kDa
WT
Δb
ΔOSCP
52
β
24.6
23
b
OSCP
33
ANT3
29
ANT2
11
g
10
e
47
CS
b
kDa
1048
720
D
M
M*
IB:β
IB:g
IB:e
c
100
HK2
47
CS
d
Calcein
TMRM
HK2pep
+CsA
e
$G_{max}$ (pS)
1000
800
600
400
200
$G_{mean}$ (pS)
$Q_{4s}$ (pC)
70
60
50
40
30
20
10

Supplementary Figure 4. **Characterization of Δb and ΔOSCP HAP1 cells. a** Western blot analysis of mitochondria isolated from WT, Δb and ΔOSCP HAP1 cells for β, b, OSCP, g and e subunits of F-ATP synthase, citrate synthase (CS), ANT2 and ANT3. The image is representative of three independent blots. **b** Clear Native-PAGE analysis and subsequent immunoblotting against subunit β, g and e of WT, Δb and ΔOSCP HAP1 mitochondria treated with 1.5 g/g digitonin. Images are representative of two independent blots. **c** Western blot analysis from isolated mitochondria of indicated genotypes for HK2, CS is used as internal control. The image is representative of three independent blots. **d** Fluorescence images of the indicated HAP1 cells loaded with 20 nM tetramethylrhodamine methyl ester (TMRM) and 500 nM calcein-AM in combination with 8 mM $CoCl_2$ before and after addition of 15 µM HK2 peptide (pep) without or with preincubation with 4 µM cyclosporin A (CsA). Bar, 40 µm. Figures are representative for data showed in Fig. 3a,b. **e** Histograms representing the maximal ($G_{max}$) and mean conductance ($G_{mean}$) and the net charge flowing through a stable open channel in an interval of 4 s ($Q_{4s}$) for the currents recorded. Only current traces with events out of the noise range were included in the analysis of $G_{mean}$. Data are average ± SEM of seven (WT and Δb) or eight (ΔOSCP) independent recordings. Source data are provided as Source Data file.

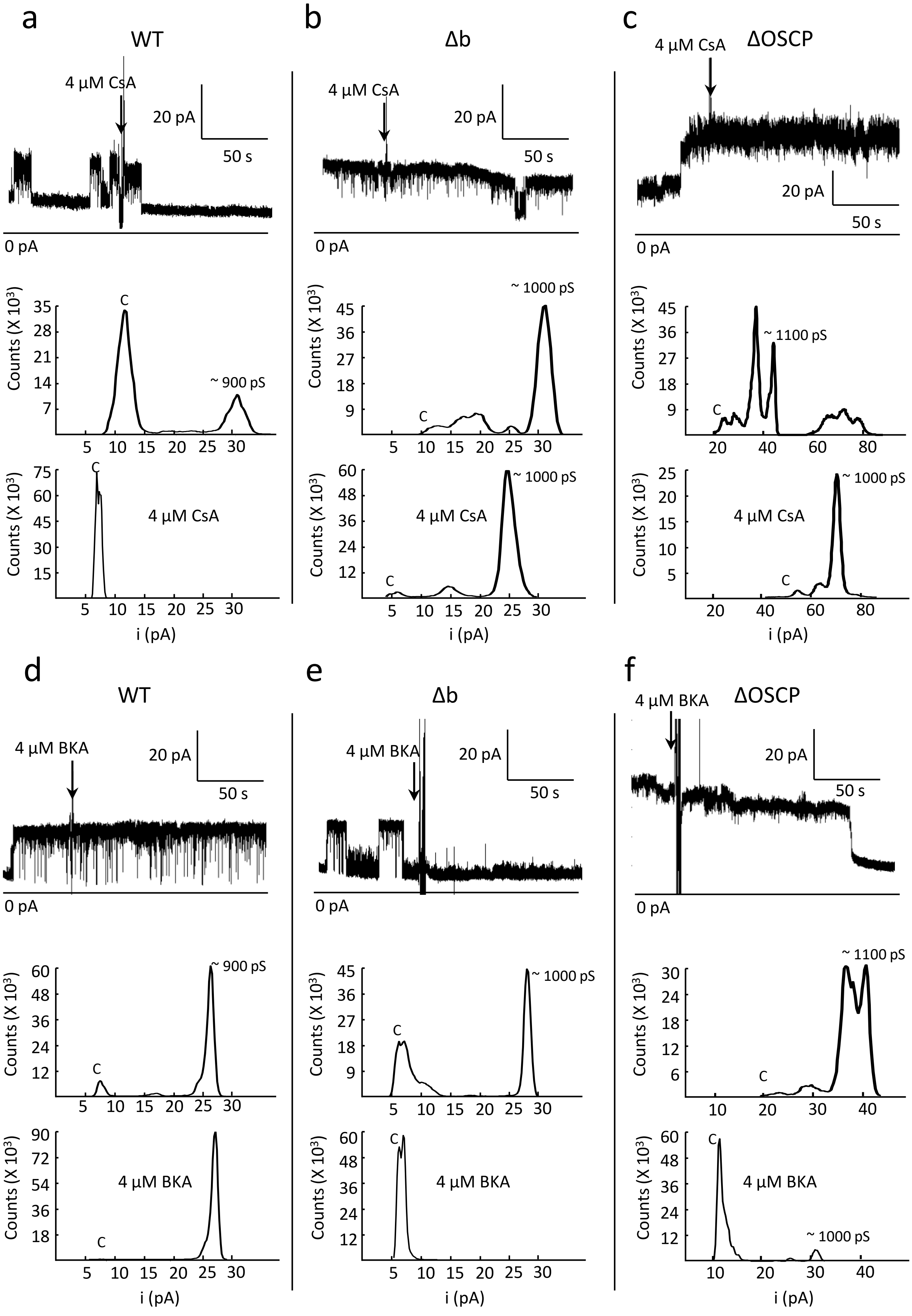
a
WT
4 μM CsA
20 pA
50 s
0 pA
Counts (X 10³)
~ 900 pS
4 μM CsA
i (pA)
b
Δb
~ 1000 pS
c
ΔOSCP
~ 1100 pS
d
4 μM BKA
e
f
C

Supplementary Figure 5. **Current analysis of Δb and ΔOSCP HAP1 mitoplasts**. **a-f** Extended (200 sec) current traces from which the recordings of Fig. 3c and 4c were obtained. Where indicated, 4 μM CsA (a-c) or 4 μM BKA (d-f) were added. Analysis of current amplitude was performed over 60 seconds starting 70 sec before and 120 sec after addition of CsA or BKA; c, closed. Source data are provided as Source Data file.

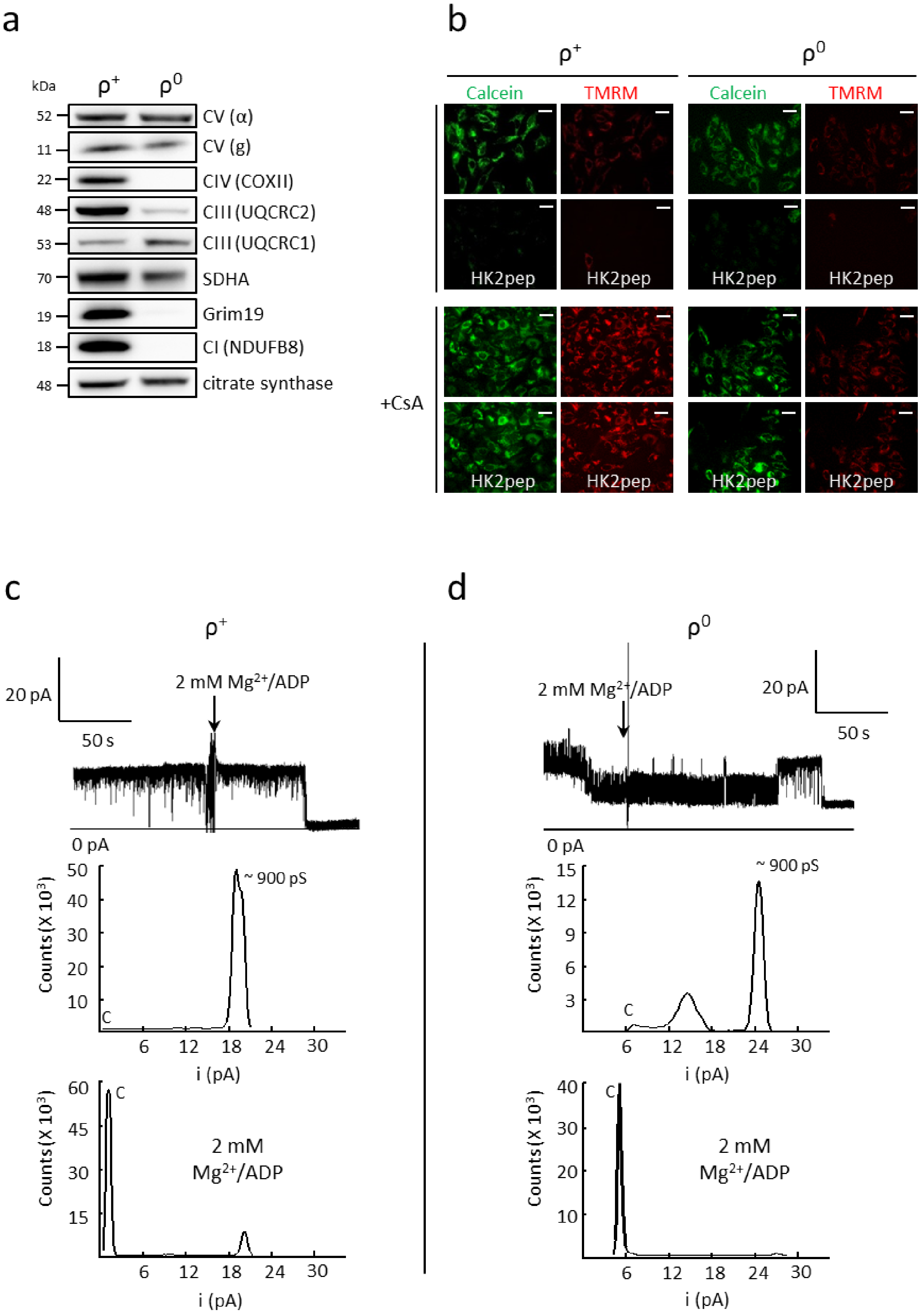
a
kDa
ρ+
ρ0
52
11
22
48
53
70
19
18
48
CV (α)
CV (g)
CIV (COXII)
CIII (UQCRC2)
CIII (UQCRC1)
SDHA
Grim19
CI (NDUFB8)
citrate synthase
b
ρ+
ρ0
Calcein
TMRM
HK2pep
+CsA
c
ρ+
20 pA
50 s
2 mM Mg2+/ADP
0 pA
~ 900 pS
Counts (X 10³)
C
i (pA)
2 mM
Mg2+/ADP
d
ρ0
2 mM Mg2+/ADP
20 pA
50 s
0 pA
~ 900 pS
Counts (X 10³)
C
i (pA)
2 mM
Mg2+/ADP

Supplementary Figure 6. **Analysis of inhibitor sensitivity of $Ca^{2+}$-dependent channel activity of $\rho^+$ and $\rho^0$ cells**. **a** Western Blot analysis of oxidative phosphorylation proteins and citrate synthase in mitochondria isolated from $\rho^+$ and $\rho^0$ cells. Images are representative of two independent blots. **b** Fluorescence images of $\rho^+$ and $\rho^0$ cells loaded with 20 nM TMRM and 500 nM calcein-AM/8 mM $CoCl_2$ before and 9 minutes after addition of 2.5 µM HK2 peptide (pep) in the absence (top panels) or presence (bottom panels) of 4 µM CsA. Bar, 40 µm. Images are representative of data showed in Fig. 5a,b. **c,d** Extended (200 sec) current traces from which recordings of Fig. 5c were obtained. Where indicated, 2 mM $Mg^{2+}$/ADP was added. Analysis of current amplitude was performed over 60 seconds starting 70 sec before and at least 40 sec after $Mg^{2+}$/ADP addition. Source data are provided as Source Data file.

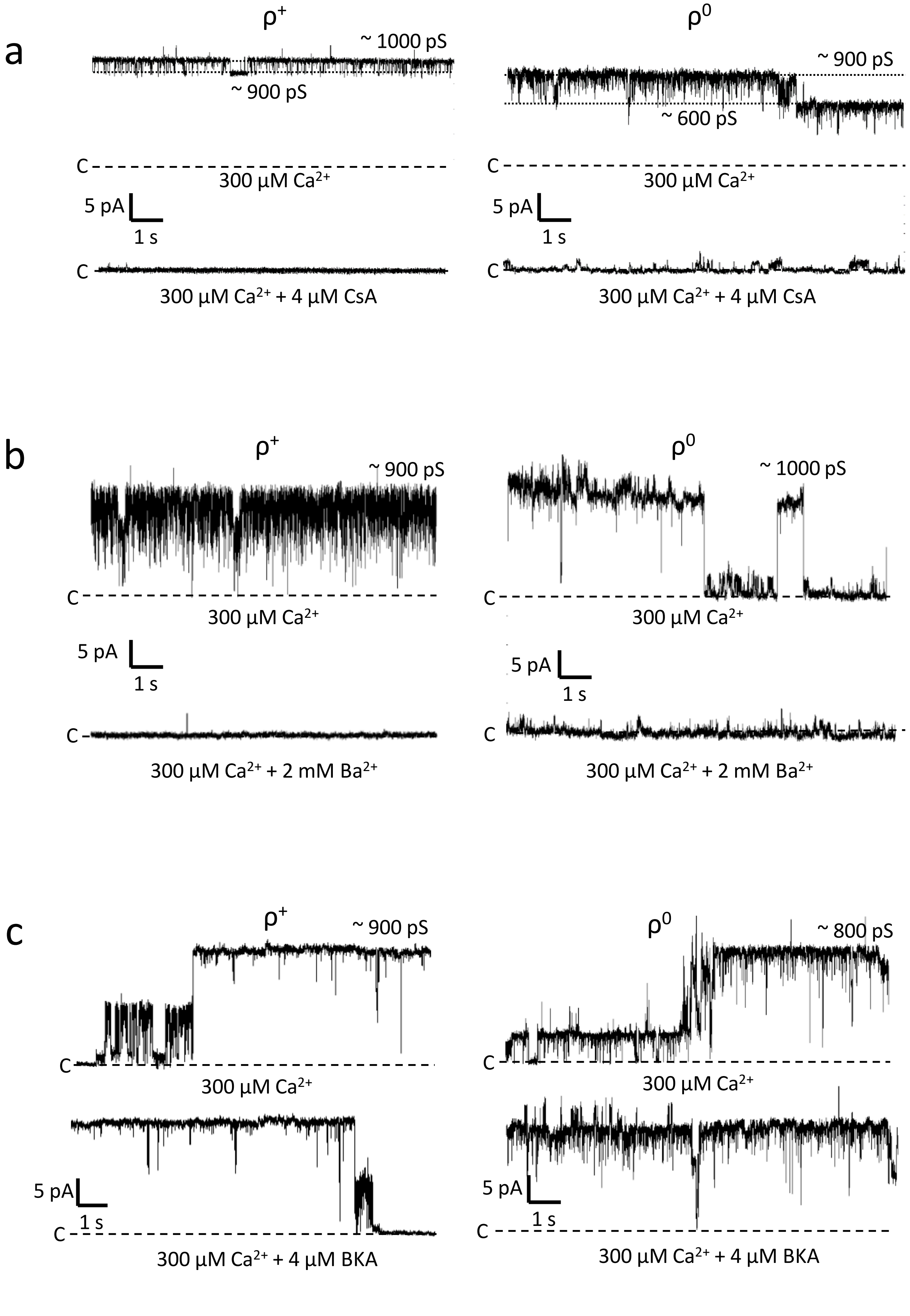
a
ρ+
~ 1000 pS
~ 900 pS
C
300 µM Ca2+
5 pA
1 s
C
300 µM Ca2+ + 4 µM CsA
ρ0
~ 900 pS
~ 600 pS
C
300 µM Ca2+
5 pA
1 s
C
300 µM Ca2+ + 4 µM CsA
b
ρ+
~ 900 pS
C
300 µM Ca2+
5 pA
1 s
C
300 µM Ca2+ + 2 mM Ba2+
ρ0
~ 1000 pS
C
300 µM Ca2+
5 pA
1 s
C
300 µM Ca2+ + 2 mM Ba2+
c
ρ+
~ 900 pS
C
300 µM Ca2+
5 pA
1 s
C
300 µM Ca2+ + 4 µM BKA
ρ0
~ 800 pS
C
300 µM Ca2+
5 pA
1 s
C
300 µM Ca2+ + 4 µM BKA

Supplementary Figure 7. **Current analysis of $\rho^+$ and $\rho^0$ mitoplasts. a-c** Representative current traces showing PTP channel activity obtained by patch-clamping isolated mitoplast from $\rho^+$ and $\rho^0$ cells in the presence of 300 µM $Ca^{2+}$($V_h$ = +20 mV) and of CsA, $Ba^{2+}$ or BKA. Source data are provided as Source Data file.

**Supplementary Table 1. gRNAs used for the CRISPR-Cas9-mediated ablation of subunit g.**

| | |
|---|---|
| gRNA 1 | TAACCTTGTGGAGAAGACCC |
| gRNA 2 | CTTGGCGTAGTACCAAAATG |

## References


1. Spikes, T. E., Montgomery, M. G. & Walker, J. E. Structure of the dimeric ATP synthase from bovine mitochondria. *Proc. Natl. Acad. Sci. USA.* **117**, 23519-23526 (2020).
