# Supplementary material for: Defining the molecular mechanisms of the mitochondrial permeability transition through genetic manipulation of F-ATP synthase
Source: Nat Commun. 2021 Aug 10;12:4835. doi: 10.1038/s41467-021-25161-x (PMC8355262; doi:10.1038/s41467-021-25161-x)
Supplement: Supplementary file 3 — Reporting summary. [file 41467_2021_25161_MOESM3_ESM.pdf]

nature research

Corresponding author(s): Paolo Bernardi, Michela Carraro

Last updated by author(s): Jul 19, 2021

Reporting Summary

Nature Research wishes to improve the reproducibility of the work that we publish. This form provides structure for consistency and transparency in reporting. For further information on Nature Research policies, see our Editorial Policies and the Editorial Policy Checklist.

Statistics

For all statistical analyses, confirm that the following items are present in the figure legend, table legend, main text, or Methods section.

n/a	Confirmed	
☐	☒	The exact sample size (*n*) for each experimental group/condition, given as a discrete number and unit of measurement
☐	☒	A statement on whether measurements were taken from distinct samples or whether the same sample was measured repeatedly
☐	☒	The statistical test(s) used AND whether they are one- or two-sided<br>*Only common tests should be described solely by name; describe more complex techniques in the Methods section.*
☒	☐	A description of all covariates tested
☒	☐	A description of any assumptions or corrections, such as tests of normality and adjustment for multiple comparisons
☐	☒	A full description of the statistical parameters including central tendency (e.g. means) or other basic estimates (e.g. regression coefficient) AND variation (e.g. standard deviation) or associated estimates of uncertainty (e.g. confidence intervals)
☐	☒	For null hypothesis testing, the test statistic (e.g. *F*, *t*, *r*) with confidence intervals, effect sizes, degrees of freedom and *P* value noted<br>*Give P values as exact values whenever suitable.*
☒	☐	For Bayesian analysis, information on the choice of priors and Markov chain Monte Carlo settings
☒	☐	For hierarchical and complex designs, identification of the appropriate level for tests and full reporting of outcomes
☒	☐	Estimates of effect sizes (e.g. Cohen's *d*, Pearson's *r*), indicating how they were calculated

Our web collection on statistics for biologists contains articles on many of the points above.

Software and code

Policy information about availability of computer code

Data collection	pClamp8.0 (Molecular Devices), LAS AF (Leica), Seahorse Wave Software
Data analysis	Clampfit (Molecular Devices), Origin, Excel, ImageJ, MATLAB (MathWorks)

For manuscripts utilizing custom algorithms or software that are central to the research but not yet described in published literature, software must be made available to editors and reviewers. We strongly encourage code deposition in a community repository (e.g. GitHub). See the Nature Research guidelines for submitting code & software for further information.

Data

Policy information about availability of data

All manuscripts must include a data availability statement. This statement should provide the following information, where applicable:

- Accession codes, unique identifiers, or web links for publicly available datasets
- A list of figures that have associated raw data
- A description of any restrictions on data availability

The data that support the findings of this study are available from the corresponding authors

Field-specific reporting

Please select the one below that is the best fit for your research. If you are not sure, read the appropriate sections before making your selection.

☒ Life sciences ☐ Behavioural & social sciences ☐ Ecological, evolutionary & environmental sciences

For a reference copy of the document with all sections, see nature.com/documents/nr-reporting-summary-flat.pdf

Life sciences study design

All studies must disclose on these points even when the disclosure is negative.

Sample size	The sample size was large enough to achieve statistical significance; individual replicate numbers and proper statistical analysis is specified in the text
Data exclusions	No data were excluded
Replication	All experiments were performed at least three times (numbers of replicates are reported in the figure legends) and replications were successful
Randomization	Randomization was not applied because all samples were treated in the same way. For electrophysiology, we did paired recordings before and after treatment for data analysis.
Blinding	Blinding was not applicable to our study. All the responses cannot be "blinded" because the activity is either there or absent.

Reporting for specific materials, systems and methods

We require information from authors about some types of materials, experimental systems and methods used in many studies. Here, indicate whether each material, system or method listed is relevant to your study. If you are not sure if a list item applies to your research, read the appropriate section before selecting a response.

Materials & experimental systems

n/a	Involved in the study	
☐	☒	Antibodies
☐	☒	Eukaryotic cell lines
☒	☐	Palaeontology and archaeology
☒	☐	Animals and other organisms
☒	☐	Human research participants
☒	☐	Clinical data
☒	☐	Dual use research of concern

Methods

n/a	Involved in the study	
☒	☐	ChIP-seq
☒	☐	Flow cytometry
☒	☐	MRI-based neuroimaging

Antibodies

Antibodies used	anti-β (ab14730, abcam), anti-gamma (PA5-29975, ThermoFisher), anti-b (ab117991, abcam), anti-OSCP (ab110276, abcam), anti-f (ab200715, abcam), anti-g (ab126181, abcam), anti-e (ab122241, abcam), anti-c (ab181243, abcam), anti-a (ab192423, abcam), anti-citrate synthase (ab96600, abcam), anti-vinculin (V4505, Sigma), anti-prohibitin (MS-261-P1, NeoMarkers), anti-GAPDH (2118, Cell Signaling) and anti-CyPD (ab110324, abcam), anti-ANT2 (14671, Cell Signaling), anti-ANT3 (PA5-35113, ThermoFischer), anti-OXPHOS (ab110411, abcam), anti-HKII (sc130358), anti UQCRC1 (sc65238, Santa Cruz), anti-Grim19 (sc271013, Santa Cruz), anti-SDHA (sc166947, Santa Cruz)
Validation	All antibodies used are commercially available, were validated by Companies by target specificity verification and functional application validation.

Eukaryotic cell lines

Policy information about cell lines

Cell line source(s)	The human 143B osteosarcoma (ρ+) and the derived 206 (ρ0) cell lines were kindly provided by Lodovica Vergani (Department of Neurosciences, University of Padova, Padova, Italy); HAP1 cells were kindly provided by Sir John Walker (Medical Research Council Mitochondrial Biology Unit, University of Cambridge, CB2 0XY Cambridge, United Kingdom); HeLa cells were purchased by ATCC (CCL-2).
Authentication	Western blotting

Mycoplasma contamination	All cell lines tested negative for mycoplasma contamination
Commonly misidentified lines (See ICLAC register)	No commonly misidentified cell lines were used in the study.
